# Peptide Binding Properties of the Three PDZ Domains of Bazooka (*Drosophila Par-3*)

**DOI:** 10.1371/journal.pone.0086412

**Published:** 2014-01-22

**Authors:** Cao Guo Yu, Raffi Tonikian, Corinna Felsensteiner, Jacquelyn R. Jhingree, Darrell Desveaux, Sachdev S. Sidhu, Tony J. C. Harris

**Affiliations:** 1 Department of Cell & Systems Biology, University of Toronto, Toronto, Ontario, Canada; 2 Terrence Donnelly Centre for Cellular and Biomolecular Research, and Banting and Best Department of Medical Research, and Department of Molecular Genetics, University of Toronto, Toronto, Ontario, Canada; 3 Centre for the Analysis of Genome Evolution & Function, University of Toronto, Toronto, Ontario, Canada; Institut Pasteur, France

## Abstract

The Par complex is a conserved cell polarity regulator. Bazooka/Par-3 is scaffold for the complex and contains three PDZ domains in tandem. PDZ domains can act singly or synergistically to bind the C-termini of interacting proteins. Sequence comparisons among *Drosophila* Baz and its human and *C. elegans* Par-3 counterparts indicate a divergence of the peptide binding pocket of PDZ1 and greater conservation for the pockets of PDZ2 and PDZ3. However, it is unclear whether the domains from different species share peptide binding preferences, or if their tandem organization affects their peptide binding properties. To investigate these questions, we first used phage display screens to identify unique peptide binding profiles for each single PDZ domain of Baz. Comparisons with published phage display screens indicate that Baz and *C. elegans* PDZ2 bind to similar peptides, and that the peptide binding preferences of Baz PDZ3 are more similar to *C. elegans* versus human PDZ3. Next we quantified the peptide binding preferences of each Baz PDZ domain using single identified peptides in surface plasmon resonance assays. In these direct binding studies, each peptide had a binding preference for a single PDZ domain (although the peptide binding of PDZ2 was weakest and the least specific). PDZ1 and PDZ3 bound their peptides with dissociation constants in the nM range, whereas PDZ2-peptide binding was in the µM range. To test whether tandem PDZ domain organization affects peptide binding, we examined a fusion protein containing all three PDZ domains and their normal linker regions. The binding strengths of the PDZ-specific peptides to single PDZ domains and to the PDZ domain tandem were indistinguishable. Thus, the peptide binding pockets of each PDZ domain in Baz are not obviously affected by the presence of neighbouring PDZ domains, but act as isolated modules with specific *in vitro* peptide binding preferences.

## Introduction

Cell polarity is fundamental to cell biology. For a cell to migrate directionally, constrict apically, transport material vectorally, or divide asymmetrically, distinguishing one end of the cell from the other is essential [1], [2], [3], [4], [5]. The Par complex is a core polarity regulator [6], [7], [8], [9]. It is made up of the adaptor protein Par-6, atypical protein kinase C (aPKC), and the scaffold protein Bazooka (Baz)/Par-3. The complex is conserved across animals, and functions in epithelial cell polarity, epithelial morphogenesis, asymmetric cell division, axon outgrowth, and cancer progression.

**Figure 1:**
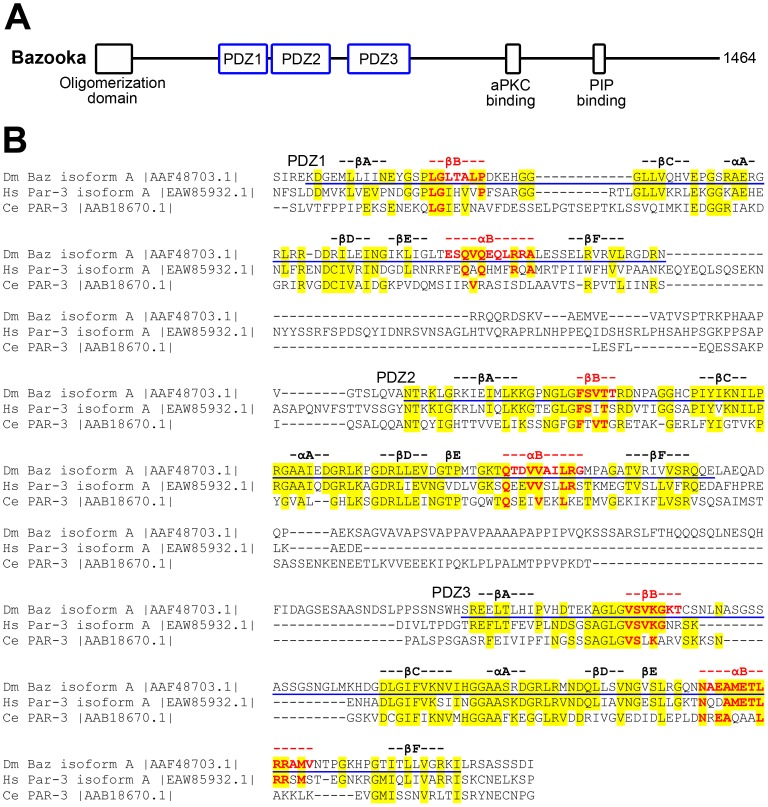
Sequence comparisons of the PDZ domains of Baz with those of human and *C. elegans* Par-3. (**A**) The overall domain organization of Baz. (**B**) Sequence alignment of the three PDZ domain tandems of Baz, human Par-3 and *C. elegans* Par-3. As determined previously [25], [26], [27], the predicted PDZ domains of Baz are underlined in blue, and predicted β-strands and α-helices in the Baz domains are indicated above the alignment. Strand β-B and helix α-B that form the peptide binding pockets of PDZ domains are in red. The initial alignment was generated by the CLUSTAL O (1.2.0) multiple sequence alignment tool. For *C. elegans* PDZ1, the alignment was further refined by determining the boundaries of the PDZ domain with InterProScan 5, and by predicting the secondary structure of the domain with Jpred3, SOPMA, HNN and Prof. Identical amino acid residues are only indicated (in yellow) within the predicted boundaries of the Baz PDZ domains. Identical residues within strand β-B and helix α-B are additionally indicated in red.

Similar to its counterparts in other species, *Drosophila* Baz contains an N-terminal oligomerization domain, three PDZ (postsynaptic density 95, discs large, zonula occludens-1) domains, a C-terminal aPKC binding region, and a C-terminal lipid binding region (Fig. 1A). *In vivo* structure-function analyses have revealed that multiple domains in Baz can act redundantly to localize the protein, and that specific domains are important for Baz activity as a polarity protein [10], [11], [12].

PDZ domains are configured to bind the C-termini of protein interaction partners [13], [14], [15]. Specifically, the PDZ domain is formed from six β-strands (βA–F) and two α-helices (αA–B). The peptide binding pocket is formed between strand βB and helix αB. Natural protein interactions involving this site occur with K_D_ values the low µM range. Within the C-termini of targets, binding specificity is strongly influenced by the C-terminal amino acid residue (position P_0_) and the second internal amino acid residue (position P_–2_).

Specific interaction partners have been found to bind to the PDZ domains of Baz and its counterparts in other species. For Baz, whole embryo lysate pull-down assays showed that Par-6 can bind to PDZ1 [16], and direct interactions have been shown between the isolated full length proteins [17]. In yeast two-hybrid analyses, aPKC and PTEN2 have been shown to interact with the second and third PDZ domains of Baz in tandem [18], [19]. Using *in vitro* binding assays, purified C-terminal portions of the adherens junction components Echinoid and Armadillo (*Drosophila* β-catenin) have been shown to bind a purified fusion protein containing all three PDZ domains of Baz in tandem [20]. Also, a purified fusion protein of the three PDZ domains of Baz has been shown to pull down a specific isoform of the atypical cadherin Flamingo from wing imaginal disk extracts [21].

Several questions arise about protein interactions with the PDZ domains of Baz. First, how conserved would such interactions be among Baz and its homologs? To cross-reference polarity networks across species, it would be useful to predict such conservation for known interactions and those yet to be discovered. Second, what are the connections between the C-termini of identified interaction partners and specific PDZ peptide binding pockets of Baz? Current interaction data mostly involves PDZ domains in tandem and thus the specific PDZ domain involved is unclear. Third, does the tandem arrangement of the Baz PDZ domains affect the binding properties of individual PDZ domains? In some proteins, such as Syntenin and Glutamate receptor interacting protein 1, neighbouring PDZ domains can affect each other’s folding and peptide binding properties [22], [23], [24], and like Baz/Par-3, many scaffold proteins contain multiple PDZ domains in tandem 13,14.

Here, we investigated these questions by first using each PDZ domain of Baz to screen for peptide interactions. Identification of these peptides allowed comparisons with peptides previously shown to bind the PDZ domains of human and *C. elegans* Par-3. Additionally, we performed quantitative binding studies between purified peptides and purified Baz PDZ domains, either alone or in tandem. These results revealed the peptide binding preferences of the Baz PDZ domains and that their peptide binding pockets act autonomously when the PDZ domains are arranged in tandem.

## Results

### The Peptide Binding Pockets of Baz PDZ2 and PDZ3 are more Conserved than that of PDZ1

To compare potential peptide binding properties of Baz and its homologs from humans and *C. elegans*, we aligned their three tandem PDZ domains in primary sequence comparisons (Fig. 1B). Additionally, we localized the peptide binding pockets of Baz within the alignment as strand βB and helix αB of each PDZ domain, as previously predicted in tertiary and secondary structure analyses [25], [26], [27]. Several features are notable within the alignment. First, Baz PDZ1 was the least conserved, both overall and at its binding pocket, and sequence identity with the *C. elegans* domain was especially poor. Second, although overall and binding pocket conservation was greater for both PDZ2 and PDZ3, the sequence identity was again greater with the human versus the *C. elegans* domains. Third, just after strand βB of Baz PDZ3 there is an extra 15–17 amino acid residues not found in the human or *C. elegans* domains. Similarly, just after strand βB of the *C. elegans* PDZ1 domain there is an extra 9–12 amino acid residues not found in the human or *Drosophila* domains. Finally, the linker regions between the PDZ domains vary in length and in sequence among the three proteins.

These sequence comparisons raised more specific questions. Does Baz PDZ1 bind different partners in the three species? Are the peptide binding preferences of Baz PDZ2 and Baz PDZ3 more similar to the human domains than the *C. elegans* domains? Could the unique stretch of amino acids found next to the peptide binding pocket of Baz PDZ3 distinguish its binding properties? If neighbouring PDZ domains affect each others’ binding preferences, would these effects be distinct in the three proteins?

### Identification of Peptide Interactions for each PDZ Domain of Baz

**Figure 2:**
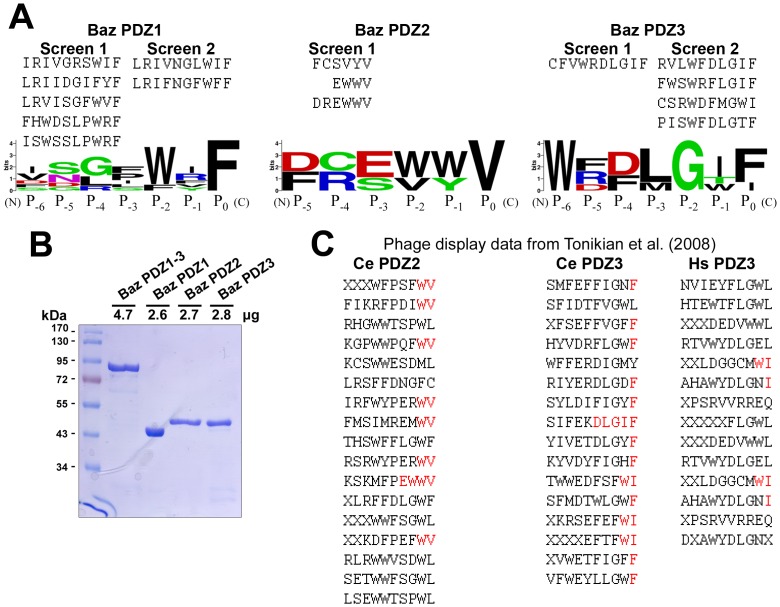
The peptide binding preferences of each PDZ domain of Baz determined by phage display screens. (**A**) Peptides identified to interact with the PDZ domains of Baz. For PDZ1 and PDZ3 screens 1 and 2 were fully independent. Individual peptides are shown with their C-termini to the right. Sequence logos with weighted amino acid positions are shown below. Colours represent the following: black, hydrophobic; green; polar; red, negative; blue positive. (**B**) A Coomassie brilliant blue-stained 10% SDS-PAGE gel showing that each GST fusion protein used in the phage display had a similar stability. Equimolar amounts were loaded. The gel also shows that GST-Baz PDZ1-3 was equally stable (further analyses shown in Table 1). (**C**) Peptides previously identified to interact with *C. elegans* Par-3 PDZ2 and PDZ3 and human Par-3 PDZ3 [28]. Position P_0_ residues with identity to those in the corresponding Baz PDZ domain-interacting peptides are shown in red. For peptides with position P_0_ matches, matching internal residues are also indicated in red until the identity with a Baz peptide ends.

To assess the peptide binding preferences of the Baz PDZ domains directly, we used GST fusion proteins of Baz PDZ1, PDZ2 and PDZ3 as separate baits in phage display screens of peptide libraries of >10^10^ unique C-terminal sequences. The screens were performed in replicate and reproducible peptide identifications were obtained for PDZ1 and PDZ3. For PDZ2, multiple screens were attempted but only one successfully identified peptides. Based on the peptides identified, consensus C-terminal binding sequences were determined (Fig. 2A). Although, GST-PDZ2 was less effective at binding phage-displayed peptides, its protein quantity and stability were indistinguishable from PDZ1 and PDZ3 by SDS-PAGE analyses of equimolar samples (Fig. 2B).

To evaluate peptide binding preferences of each domain, we compared the identified sequences. PDZ1 reproducibly interacted with peptides with phenylalanine at their C-termini (position P_0_) and had a strong preference for tryptophan at position P_–2_ (Fig. 2A). PDZ2 bound three distinct peptides with valine at their C-termini (P_0_) (Fig. 2A). Similar to PDZ1, PDZ3 reproducibly interacted with peptides with phenylalanine at their C-termini (P_0_) but unlike PDZ1, the peptides had glycine at position P_–2_ (Fig. 2A). Despite the identity of the P_0_ position in peptides that bound the PDZ1 and PDZ3, comparing strand βB and helix αB between the domains revealed had no apparent similarities (Fig. 1). The P_–2_ position was also different in the peptide binding profiles for PDZ1 and PDZ3 suggesting it may distinguish the peptide binding properties of the domains, consistent with both positions P_0_ and P_–2_ functioning in peptide recognition [13].

To compare the peptide binding preferences of the Baz PDZ domains to those of human and *C. elegans* Par-3, we compared our dataset to those determined for *C. elegans* PDZ2 and PDZ3 and human PDZ3 [28] (Fig. 2C). For *C. elegans* PDZ2, peptide position P_0_ was also commonly valine (8/17 peptides [47%]). 15/17 peptides also matched the Baz PDZ2 peptides at position P_-1_, but only one matched at position P_–2_ (it closely matched the Baz PDZ2 peptides, ending with -EWWV). For *C. elegans* PDZ3, 14/16 position P_0_ residues (88%) matched those for Baz PDZ3 peptides, and 13/16 position P_–2_ residues (81%) matched those of Baz PDZ3 peptides (one matched very closely, ending with –DLGIF as did two Baz PDZ3 peptides). In contrast, for human PDZ3, only 4/14 position P_0_ residues (29%) matched those for Baz PDZ3 peptides, and 8/14 position P_–2_ residues (57%) matched those of Baz PDZ3 peptides. Thus, Baz PDZ2 and PDZ3 have very similar peptide binding preferences compared to the domains from *C. elegans* Par-3, and for PDZ3 (for which the comparison was possible) the binding preferences were more similar between the *Drosophila* and *C. elegans* domains versus the human domain.

### Specific and Concentration-dependent Interactions between Individual Peptides and PDZ Domains

**Figure 3:**
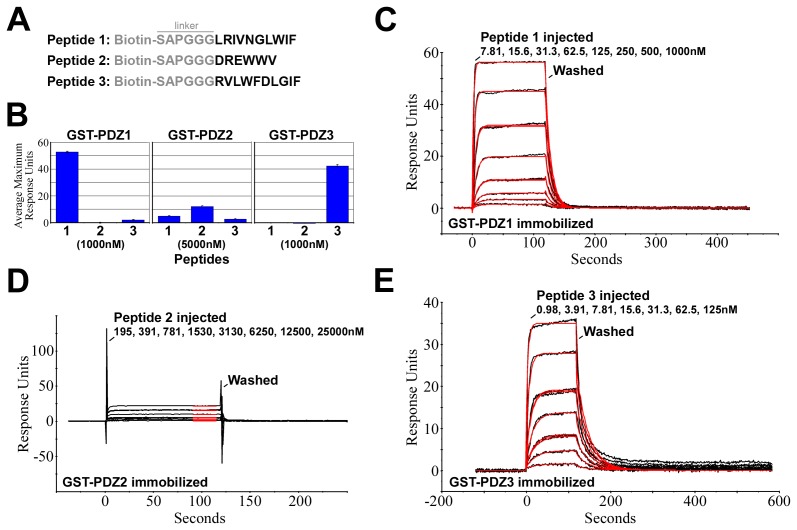
Quantification of Baz PDZ domain-peptide interactions by surface plasmon resonance. (**A**) Peptides used in direct interaction assays. They include a common linker that was also found in the peptide library for the phage display screen, and they were biotinylated at their N-termini. One peptide was selected for each of the PDZ domains from those identified in the phage display screen (Peptide 1 for PDZ1, etc). (**B**) Quantifications of binding between each peptide and each PDZ domain at the indicated peptide concentrations by SPR. PDZ1 and PDZ3 showed strong selective binding to their respective peptides. PDZ2 showed weaker binding but still preferred Peptide 2. The data represent the means±SD for four runs with the same protein and peptide samples. The experiment was replicated with separately purified proteins and separately prepared peptides with similar results (data not shown). (**C**) SPR response curves for different concentrations of Peptide 1 binding to immobilized GST-PDZ1. The peptide injection and wash times are indicated. The red lines are fits of the curves used to determine the K_D_ values shown in Table 1. (**D–E**) The equivalent analyses as in (**C**) but for Peptide 2-PDZ2 and Peptide 3-PDZ3 binding, respectively. Note the weaker interactions of Peptide 2-PDZ2.

To test the binding of purified peptides to the PDZ domain GST fusion proteins, we acquired one peptide for each PDZ domain corresponding to single peptides identified by phage display (Fig. 3A). We separately immobilized PDZ domain GST fusion proteins on solid supports and analyzed peptide binding by surface plasmon resonance (SPR).

First, we tested the binding specificity of each peptide-PDZ domain pair by quantifying the binding of all nine possible pairings (Fig. 3B). For PDZ1 and PDZ3, strong and specific binding was observed for Peptide 1 and Peptide 3, respectively. For PDZ2, higher peptide concentrations were needed to detect binding, and although preferential binding to Peptide 2 occurred, binding to Peptides 1 and 3 was also substantial. Thus, for the peptides identified from the peptide library, PDZ1 and PDZ3 had very strong and specific binding preferences, whereas PDZ2-peptide interactions appeared weaker and less specific.

Next we tested if the peptide-PDZ domain interactions were dependent on peptide concentrations. For all three pairs (PDZ1-Peptide 1, PDZ2-Peptide 2 and PDZ3-Peptide 3), the detection of binding depended on peptide concentration, keeping PDZ domain concentrations constant (Fig. 3C–E). For PDZ1-Peptide 1 and PDZ3-Peptide 3, robust binding occurred with relatively low peptide concentrations, but for PDZ2-Peptide 2 relatively high peptide concentrations were needed for binding. Since all three interactions were concentration-dependent, and reversible, we were able to determine their K_D_ values. PDZ1 and PDZ3 bound their peptides with K_D_ values in the nM range, whereas PDZ2-Peptide 2 binding was in the µM range (Table 1).

**Table 1:** Peptide binding strengths of Baz PDZ domains alone or in tandem.

Immobilized protein	Peptide partner	Replicate*	K_D_ (M)
GST-PDZ1	Biotin-SAPGGGLRIVNGLWIF	1	1.64E-07
	(*PDZ1 peptide from phage* *display*)	2	1.86E-07
GST-PDZ1-3		1	2.63E-07
		2	2.45E-07
GST-PDZ2	Biotin-SAPGGGDREWWV	1	1.77E-05
	(*PDZ2 peptide from phage* *display*)	2	1.30E-05
GST-PDZ1-3		1	9.13E-06
		2	9.98E-06
GST-PDZ3	Biotin-SAPGGGRVLWFDLGIF	1	4.59E-08
	(*PDZ3 peptide from phage* *display*)	2	2.52E-08
GST-PDZ1-3		1	4.94E-08
		2	3.26E-08

* Replicates used separately purified proteins and separately prepared peptides.

### Tandem PDZ Domain Arrangement does not Affect the Binding Strengths of Individual Peptide-PDZ Domain Interactions

With the binding strengths of individual PDZ domain-peptide interactions quantified, we next investigated whether the natural tandem arrangement of the PDZ domains affects their peptide binding. For these experiments, we purfied GST-PDZ1-3 and confirmed its stability by SDS-PAGE (Fig. 2B). To test the binding properties of the individual PDZ domains within the tandem, we separately probed GST-PDZ1-3 with Peptides 1, 2 and 3. The binding responses were concentration dependent and very similar to those of the isolated domains (data not shown). To assess binding quantitatively, we determined the K_D_ values of GST-PDZ1-3 binding to each peptide. Strikingly, the values were very close to those of each GST-PDZ domain alone (the single PDZ1-Peptide 1, PDZ2-Peptide 2 and PDZ3-Peptide 3 pairings) (Table 1). Thus, the peptide binding pockets of each PDZ domain appear to be unaffected by tandem organization of the PDZ domains.

## Discussion

Our work describes the peptide binding preferences of the three PDZ domains of Baz. Identifying these preferences allows comparisons with the PDZ domains of *C. elegans* and human Par-3, assessment of the effects of tandem PDZ arrangement, further evaluations of proteins shown to interact with Baz, and considerations of how the peptides could be used to manipulate Baz/Par-3 activities *in vivo*.

In previous phage display screens, interacting peptides were identified for PDZ2 and PDZ3 from *C. elegans* Par-3, and PDZ3 from human Par-3 [28]. The peptides identified for Baz PDZ2 are very similar to those for PDZ2 from *C. elegans*–peptides ending in -WV are common in both datasets, and the C-terminal sequence -EWWV occurs in peptides in both datasets. In comparison, Baz PDZ2, defined through 3D structures and secondary structure predictions [25], [26], [27], has 36/89 (40%) amino acid residue identity with *C. elegans* PDZ2 and 51/88 (58%) identity with human PDZ2 (using default NCBI BLAST settings). The peptides identified for Baz PDZ3 are also very similar to those for *C. elegans* PDZ3–peptides ending in -F are common in both datasets, and the C-terminal sequence -DLGIF occurs in peptides in both datasets. The overall peptide binding profile for human PDZ3 was more distinct (with no peptides ending in –F), although peptides ending with –WI were detected in the datasets for PDZ3 from all three species. The greater similarity of the peptide preferences of PDZ3 from *Drosophila* and *C. elegans* versus human was surprising as *Drosophila* PDZ3 has less overall amino acid identity with *C. elegans* PDZ3 (33/98 (34%)) versus human PDZ3 (59/116 (51%)). Although, data are not available for PDZ1 from the other species, Baz PDZ1 has a peptide-binding profile of Class 4 PDZ domains [28]. Of note, PDZ1 is the least conserved of the Baz PDZ domains (Fig. 1). Overall these data indicate that the binding properties of the PDZ domains of Baz are very similar to those of *C. elegans* Par-3 and somewhat less similar to those of human Par-3.

In some proteins, such as Syntenin and Glutamate receptor interacting protein 1, neighbouring PDZ domains can affect each other’s folding and peptide binding properties [22], [23], [24], but our quantification of peptide binding to each Baz PDZ domain alone or in tandem indicated that the peptide binding pockets of each PDZ domain are unaffected by the presence of neighbouring PDZ domains. Such independence is consistent with the ability of each individual PDZ domain to affect the *in vivo* localization of Baz constructs in which the other two PDZ domains were deleted in an otherwise intact protein [12].

No *Drosophila* protein had a C-terminus with an exact match to any of the peptides identified by phage display, consistent with the unnaturally high binding affinities of peptides identified by phage display [28]. Nonetheless, comparisons between known Baz interacting proteins and the identified peptides allow predictions of specific interaction sites. For example, Par-6 has the C-terminus –VLHL, and thus has little similarity to peptides that bind PDZ1. Possibly, the identified Par-6 interaction with Baz PDZ1 [16] involves regions outside of the peptide binding pocket, as evident for the binding of *C. elegans* Par-6 to PDZ1 of Par-3 [29]. Additionally, aPKC and PTEN2 have been shown to interact with the second and third PDZ domains of Baz in tandem [18], [19]. aPKC ends with –EDCV suggesting a direct interaction with the peptide binding pocket of PDZ2. PTEN2 ends with –STYL and only matches positions P_-1_ and P_-3_ of one peptide that bound Baz PDZ2. However, leucine was present at position P_0_ of many of the peptides that bound PDZ2 and 3 from *C. elegans* and humans. In other experiments, purified C-terminal portions of the adherens junction components Echinoid and Armadillo have been shown to bind a purified fusion protein containing the three PDZ domains of Baz [20]. At its C-terminus, Echinoid ends with –EIIV, suggesting it may directly interact with the peptide binding pocket of PDZ2. However, the interacting Arm isoform ends with –DTDC which does not match any peptides identified, and thus suggests a binding mechanism outside of the peptide binding pockets. Finally, the isoform of Flamingo that was pulled down from cell lysates with the tandem Baz PDZ1-3 protein has a C-terminus of -DDETTV [21], matching all Baz PDZ2 peptides at the P_0_ position, and two additionally at the P_-3_ position and one additionally at the P_-5_ position. As more proteins are found to interact with Baz, the peptides we have identified will hopefully aid in mapping their specific interaction sites.

The unnaturally high binding strengths of the identified peptide-PDZ interactions could be utilized for manipulating Baz *in vivo*. By SPR, we measured K_D_ values in the nM range for peptide binding to PDZ1 and PDZ3, and in the µM range for peptide binding to PDZ2. These binding strengths could allow the peptides to out-compete normal Baz interactions *in vivo*. Such competition could be used to inhibit Baz or re-wire Baz interactions. These effects could be useful for experimental manipulations, and possibly for treatment of disease. For example, the peptide binding pocket of Baz PDZ1 has been shown to promote the removal of full length Baz from the apical circumference of epithelial cells [12]. Under conditions in which cell polarity is otherwise weakened, inhibition of this Baz removal mechanism could help stabilize normal cell organization. The identified high affinity peptides for Baz PDZ1 might inhibit the removal mechanism. Since loss of Par-3 activity has been linked to tissue destabilization during mammalian cancer progression [30], [31], such mechanisms and treatment strategies are worth considering.

## Materials and Methods

### Phage Display Screen

The phage display screen used previously designed and analyzed GST fusion proteins of each PDZ domain of Baz [25]. The screen followed an established protocol [32], as performed previously for the PDZ domains of human and *C. elegans* Par-3 [28]. Briefly, each PDZ domain was separately used to screen phage particles each expressing, and displaying on their surface, one of >10^10^ unique C-terminal peptides in the library. A series of iterative panning steps enriched for strong interactions. Following this enrichment, individual PDZ domain-phage interactions were confirmed in ELISA binding assays. The peptide sequences corresponding to these phage particles were determined by DNA sequencing for the phage surface protein displaying the peptides of the library. Interactions of sibling phages (those with identical peptide sequences) were considered as a single PDZ domain-peptide interaction.

### Surface Plasmon Resonance Binding Studies

Binding experiments were conducted with a Biacore 3000 biosensor (GE Healthcare, USA), as described [33]. Specifically, the GST fusion proteins of each PDZ domain alone or of the PDZ domains in tandem were immobilized on CM5 chips (GE Healthcare) at a concentration of 30 µg/ml in 10 mM sodium acetate (pH 5.5) for PDZ1, PDZ3 and PDZ1-3, and in 10 mM HEPES (pH 7.0) for PDZ2. The peptides for SPR were biotinylated but otherwise identical to those indentified by phage display (Peptide 1, Biotin-SAPGGGLRIVNGLWIF; Peptide 2, Biotin-SAPGGGDREWWV; Peptide 3, Biotin-SAPGGGRVLWFDLGIF; synthesized by Sheldon Biotechnology Centre, McGill University, Canada). All peptides were solubilized in HBS-EP running buffer (10 mM Hepes pH 7.4, 150 mM NaCl, 3 mM EDTA, 0.005% Surfactant P20) and were flowed over the immobilized proteins in this buffer. For Peptide 1 and Peptide 3, K_D_ values were determined with a 1∶1 binding model with drifting baseline. For Peptide 2, K_D_ values were determined with a steady state model.
